# Severe and Prolonged Hypoglycemia Following Adrenalectomy for Pheochromocytoma: A Case Report

**DOI:** 10.7759/cureus.86880

**Published:** 2025-06-27

**Authors:** Masahiko Kawasumi, Yoshiro Sakamoto, Yuka Tosaka, Yukiko Okazaki, Shoko Furuya

**Affiliations:** 1 Department of Metabolism and Endocrinology, Internal Medicine, Juntendo University Nerima Hospital, Nerima, JPN; 2 Department of Urology, Juntendo University Nerima Hospital, Nerima, JPN

**Keywords:** adrenal pheochromocytoma, catecholamine excess, glucose metabolism, hypoglycemia, insulin sensitivity, postoperative hypoglycemia, secondary diabetes

## Abstract

We report a case of a 64-year-old woman with poorly controlled diabetes mellitus who was found to have a left adrenal pheochromocytoma. Despite standard preoperative management including alpha-blockade with doxazosin, the patient developed persistent and severe hypoglycemia immediately following adrenalectomy, requiring high-rate intravenous glucose infusion. Markedly suppressed preoperative urinary C-peptide levels rapidly increased after surgery, indicating reversible beta-cell suppression due to catecholamine excess. Over a 10-year postoperative course, the patient maintained stable glycemic control with dietary therapy alone, demonstrating substantial improvement in glucose metabolism. This case highlights the rare but significant risk of postoperative hypoglycemia even under optimized perioperative conditions, and illustrates the potential for long-term improvement in diabetes following pheochromocytoma resection.

## Introduction

Pheochromocytoma is a rare catecholamine-secreting tumor arising from the chromaffin cells of the adrenal medulla, characterized by excessive production of one or more catecholamines. Classic clinical features include headache, hypertension, and profuse sweating. Although these symptoms are considered hallmark signs, the clinical presentation can be variable, and in some cases, the tumor may remain undiagnosed until complications occur. The estimated prevalence is approximately 0.1-0.6% among patients with hypertension [1]. In recent years, advancements in imaging techniques have led to the increased detection of adrenal incidentalomas. According to a meta-analysis, pheochromocytoma accounts for approximately 4% of these adrenal incidentalomas [2]. When classic symptoms are present in conjunction with an adrenal mass, diagnostic specificity and sensitivity exceed 90%[3], underscoring the importance of a thorough evaluation. In addition to its cardiovascular effects, pheochromocytoma can induce metabolic disturbances, including glucose intolerance and diabetes mellitus, due to catecholamine-induced insulin resistance and suppressed insulin secretion [4,5].

We report a case of a woman with poorly controlled diabetes mellitus in whom pheochromocytoma was subsequently diagnosed. Following adrenalectomy, she developed persistent severe hypoglycemia. This case highlights the potential for postoperative hypoglycemia even under optimized preoperative management and demonstrates that beta-cell dysfunction associated with catecholamine excess may be reversible.

## Case presentation

A 64-year-old woman presented with poorly controlled diabetes mellitus, initially diagnosed at age 55, and a history of hypertension and hypercholesterolemia. Her HbA1c levels remained above 9% despite treatment with oral hypoglycemic agents, including glimepiride and metformin. Her body mass index (BMI) was 22.2 kg/m^2^, calculated from a height of 150 cm and a weight of 50 kg. She experienced episodes of hypoglycemia following sulfonylurea use, which prompted referral to our hospital. The episodes were sudden and occurred without clear provocation, sometimes developing during ordinary activities such as walking outdoors.

Her medical history included cholecystectomy at age 36 and hysterectomy at age 43. Her father had hypertension. She smoked 20 cigarettes daily and consumed alcohol occasionally. Medications included amlodipine, candesartan, glimepiride (1 mg), metformin (250 mg), and atorvastatin.

Abdominal CT previously performed by the referring physician had revealed a left adrenal mass, initially interpreted as a nonfunctioning adrenal incidentaloma. She did not report paroxysmal hypertension, headache, or abdominal pain, which are classic symptoms of pheochromocytoma. However, during clinical evaluation, we observed excessive facial sweating, which the patient considered normal for her. Based on these findings, pheochromocytoma was suspected, and further endocrine evaluation was initiated. No thyroid enlargement or abnormal heart/lung sounds were present. Blood tests revealed chronic hyperglycemia, and markedly elevated urinary catecholamine levels confirmed the diagnosis of pheochromocytoma. At the time, plasma-free metanephrine and normetanephrine testing was not covered by national insurance and therefore was not performed. These preoperative laboratory findings are summarized (Table 1).

**Table 1:** Preoperative Laboratory Data This table summarizes preoperative laboratory data. HbA1c was markedly elevated, indicating chronic hyperglycemia, while fasting blood glucose was within the normal range. C-peptide was significantly suppressed (25.8 μg/day), consistent with beta-cell suppression. Markedly elevated levels of urinary catecholamines supported the diagnosis of pheochromocytoma. Note: The fasting immunoreactive insulin (IRI) level may have been influenced by ongoing basal insulin therapy and possible assay cross-reactivity, and therefore may not accurately reflect endogenous insulin secretion.

Parameter	Value	Reference Range
Fasting blood glucose	88	65-109 mg/dL
HbA1c	9.5	4.7-6.2%
C-peptide	0.73	0.61-2.09 ng/mL
Serum insulin	14.2	1.8-12.2 μU/mL
24-hour urine C-peptide	25.8	29.2-167 μg/day
Adrenaline	1291	<100 pg/mL
Noradrenaline	3562	<500 pg/mL
Dopamine	138	<20 pg/mL
Urinary metanephrine (corrected)	11.1	<1.0 mg/g・Cr
Urinary normetanephrine (corrected)	3.95	<0.9 mg/g・Cr

She was started on alpha-blockade therapy with doxazosin at a dose of 2 mg/day as preoperative management. As part of the preoperative preparation, sulfonylureas were discontinued more than two days before surgery and replaced with a basal-bolus insulin regimen to minimize the risk of hypoglycemia and allow for more flexible glycemic control.

**Figure 1:**
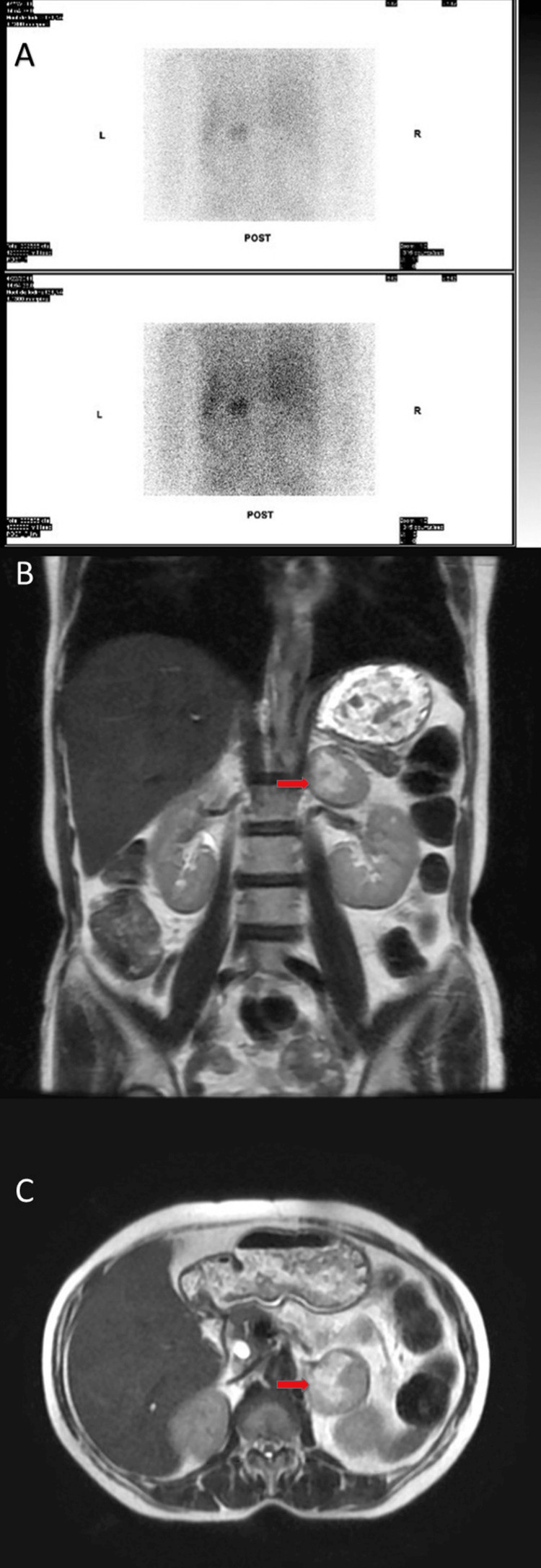
Imaging Findings Consistent With Left Adrenal Pheochromocytoma (A) Iodine-131 metaiodobenzylguanidine (^131^I-MIBG) scintigraphy showing focal radiotracer uptake in the left adrenal region, consistent with a catecholamine-secreting tumor. (B) Coronal T2-weighted MRI demonstrating a well-circumscribed, hyperintense mass in the left adrenal gland measuring approximately 4.4 cm in diameter (red arrow). (C) Axial T2-weighted MRI confirming the adrenal lesion with high signal intensity and smooth, ovoid morphology (red arrow).

Imaging with iodine-131 metaiodobenzylguanidine (^131^I-MIBG) scintigraphy and MRI revealed a left adrenal mass consistent with pheochromocytoma (Figure 1). While ^123^I-MIBG is now more commonly used due to superior imaging quality, ^131^I-MIBG was routinely employed at the time this case occurred. She underwent open left adrenalectomy. An open approach was chosen over a laparoscopic procedure due to institutional preference and surgeon expertise at the time, as laparoscopic adrenalectomy had not yet been standardized for pheochromocytoma at our center. Postoperatively, noradrenaline infusion was used to stabilize blood pressure.

**Figure 2:**
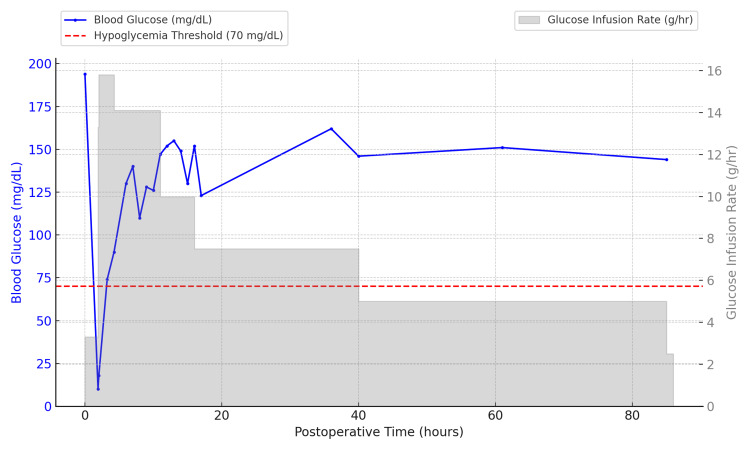
Continuous Glucose Infusion and Blood Glucose Levels After Adrenalectomy This figure illustrates the continuous glucose infusion rate (gray bars) and corresponding blood glucose levels (blue line) in a patient following adrenalectomy. The red dashed line represents the hypoglycemia threshold (70 mg/dL). The glucose infusion rate is depicted as a continuous, uninterrupted administration, accurately reflecting the clinical course.

Upon return to the general ward after surgery, her blood glucose dropped to 18 mg/dL, prompting administration of an intravenous bolus of approximately 13 grams of glucose. Due to the severity of the hypoglycemia and the need for frequent monitoring and high-rate intravenous glucose infusion, she was transferred to the ICU. Her glucose levels gradually stabilized, and she was discharged from the ICU approximately 12 hours later (Figure 2).

**Figure 3:**
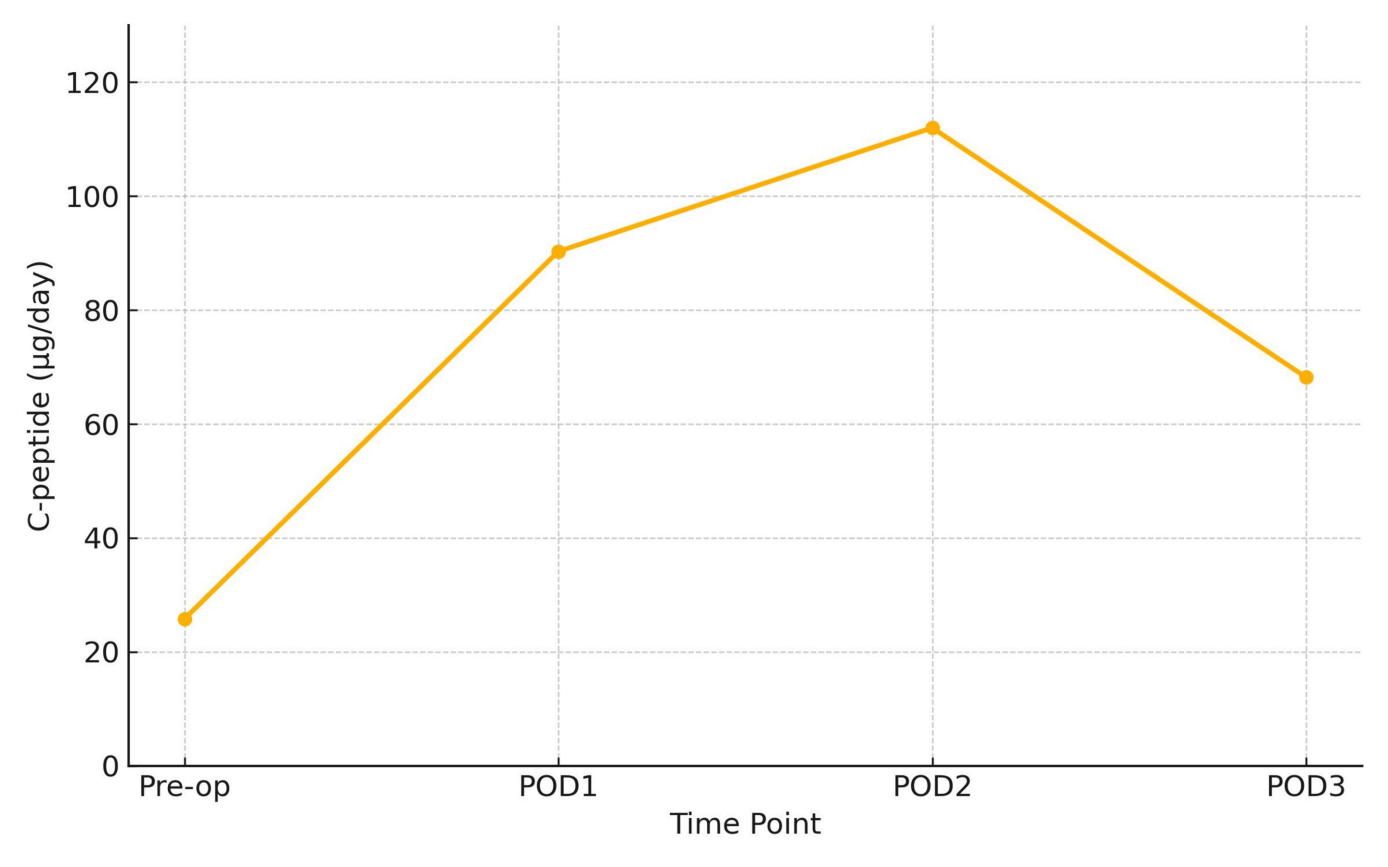
Postoperative Changes in 24-Hour Urine C-peptide Excretion Serial measurements of 24-hour urinary C-peptide levels before and after adrenalectomy. The preoperative value was markedly decreased (25.8 μg/day; reference range: 29.2-167 μg/day), suggesting suppressed endogenous insulin secretion likely due to catecholamine excess. A rapid postoperative increase was observed, peaking on postoperative day 2 (112.0 μg/day), followed by gradual stabilization. This trend supports the reversibility of beta-cell suppression in pheochromocytoma-associated secondary diabetes.

Urinary C-peptide levels rose markedly (Figure 3), indicating endogenous hyperinsulinemia. On the day of hypoglycemia, the patient’s serum cortisol was 17.3 μg/dL (reference range: 4.0-18.3), adrenocorticotropic hormone (ACTH) was 90.3 pg/mL (7.2-63.3), and serum sodium was 141 mmol/L (135-145), with no findings suggestive of adrenal insufficiency. By five months postoperatively, without any hypoglycemic therapy, her HbA1c had improved to 6.0%. The patient’s body weight fluctuated mildly over the years, increasing from 50 kg preoperatively to 54 kg at 10 years postoperatively, with an overall trend toward gradual weight gain. Long-term glycemic control without pharmacological intervention is summarized (Table 2). She has remained normoglycemic on dietary therapy alone for over 10 years.

**Table 2:** Postoperative Follow-Up Data Follow-up data after adrenalectomy showing progressive improvement in glycemic control without the need for antidiabetic medications. HbA1c and fasting glucose remained stable over a 10-year period.

Time Point	HbA1c (%)	Glucose (mg/dL)	Treatment
1 month postop	7.8	134	No medication
3 months postop	6.9	89	No medication
5 months postop	6.6	99	No medication
12 months postop	6.2	95	No medication
10 years postop	6.8	112	No medication
Reference range	4.7-6.2	65-109	

## Discussion

Postoperative hypoglycemia following adrenalectomy for pheochromocytoma, while historically reported with an incidence as high as 12-43%, has become less common in recent years due to improved perioperative management [6,7]. A retrospective study by Yamanashi et al. reported no cases of postoperative hypoglycemia after the introduction of careful alpha-blockade protocols with doxazosin [7]. Nonetheless, risk is not uniformly eliminated, particularly in patients with predisposing factors such as large tumor size, high catecholamine output, and poorly controlled diabetes mellitus [8].

In our case, the mechanism of postoperative hypoglycemia appears to involve rebound hyperinsulinemia due to abrupt withdrawal of chronic catecholamine-mediated beta-cell suppression, particularly via α2-adrenergic receptors. This mechanism was originally detailed by Young and Landsberg in their review on catecholamines and metabolic regulation [9]. The release of suppression unmasks residual beta-cell function, leading to excessive endogenous insulin secretion. Our patient’s fourfold increase in urinary C-peptide following surgery substantiates this mechanism, consistent with previous findings reported by Akiba et al. [10], who documented similar postoperative elevations in patients with catecholamine-induced beta-cell suppression.

While improved insulin sensitivity postoperatively has been proposed, its contribution appears limited. Komada et al. showed modest changes in insulin resistance after surgery, with rebound insulin secretion being the dominant driver of hypoglycemia in the acute phase [11]. In our case, insulin assays such as homeostatic model assessment (HOMA) indices were not conducted, but the acute timing, severity, and biochemical profile point toward a secretion-based mechanism.

Importantly, the patient had previously experienced unexplained hypoglycemic episodes while on sulfonylurea therapy. These unpredictable drops in blood glucose, even during routine activities, suggested intermittent release from catecholamine-mediated beta-cell suppression. This clinical observation, in retrospect, implies preserved beta-cell capacity that was functionally inhibited before surgery.

Although dynamic insulin testing was not performed, our case supports that a preserved insulin reserve under catecholaminergic suppression may manifest as severe hypoglycemia once suppression is removed. Preoperative indicators such as body composition, duration of diabetes, and history of sulfonylurea-associated or paradoxical hypoglycemia may help stratify risk for this rare but significant complication.

Given the potential for such rebound responses, even in patients with preexisting diabetes, clinicians should maintain heightened vigilance during the perioperative period. Although this is a single case, the findings may offer insight into a broader pathophysiological mechanism relevant to similar clinical scenarios.

Additionally, this patient maintained long-term remission from diabetes, over 10 years, on dietary therapy alone. This outcome suggests that catecholamine-induced beta-cell dysfunction in pheochromocytoma is not only reversible but may result in durable glycemic normalization, especially in patients with lower BMI and shorter diabetes duration. Beninato et al. reported that preoperative BMI, rather than tumor size, was an independent predictor of diabetes remission post-adrenalectomy [12].

Clinicians should continue to monitor for hypoglycemia in the immediate postoperative phase, particularly in non-obese patients or those with paradoxical glycemic fluctuations before surgery. This case illustrates both the risks and opportunities associated with resection of pheochromocytoma and reinforces the importance of individualized perioperative planning and long-term follow-up.

## Conclusions

This case highlights the potential for prolonged severe hypoglycemia after adrenalectomy for pheochromocytoma, even with appropriate preoperative preparation. The patient’s clinical course underscores the importance of individualized perioperative risk assessment, especially in those with preexisting diabetes and elevated catecholamine levels. Moreover, this case demonstrates the potential for significant improvement in glucose metabolism following tumor resection, highlighting the reversible nature of catecholamine-induced beta-cell dysfunction. Careful perioperative glucose monitoring and long-term follow-up are essential in optimizing outcomes in such cases.
